# Removable brace as a viable alternative to cast immobilization for ankle fractures—a meta-analysis of randomized controlled trials

**DOI:** 10.3389/fmed.2025.1594505

**Published:** 2025-07-29

**Authors:** Xi Li, Kaiwen Yi, Shiqi Li, Na Liu, Lemei Zhu

**Affiliations:** ^1^Hunan Key Laboratory of the Research and Development of Novel Pharmaceutical Preparations, Changsha Medical University, Changsha, China; ^2^The Hunan Provincial University Key Laboratory of the Fundamental and Clinical Research on Functional Nucleic Acid, Changsha, China; ^3^School of Public Health, Changsha Medical University, Changsha, China; ^4^Department of Cardiovascular Medicine, The Second Xiangya Hospital of Central South University, Changsha, China

**Keywords:** ankle fractures, removable brace, cast immobilization, meta-analysis, weight bearing

## Abstract

**Background:**

Recent years have seen continuous debate over the preferred method of immobilization for ankle fractures, especially between removable braces and cast immobilization. To address this, we conducted a meta-analysis of prospective randomized controlled trials (RCTs) to compare the two approaches and assess the feasibility of using a removable brace as an alternative to cast immobilization.

**Methods:**

PubMed, Cochrane Library, Embase, and Web of Science were last searched on January 18, 2025, to identify comparative studies evaluating removable braces vs. cast immobilization. Data were extracted and pooled, and a meta-analysis was conducted using Review Manager 5.4 (RevMan 5.4), The Cochrane Collaboration. Functional scores, complications, and time to return to work (RTW) were analyzed to assess the efficacy, safety, and cost of the two groups.

**Results:**

We included 11 RCTs with a total sample size of 1,472 participants. There were no significant differences in the Olerud–Molander Ankle Score (OMAS) between the removable brace and cast immobilization groups, both in the short term at 6 weeks [mean differences (MD): 7.18, 95% confidence interval (CI): −5.77 to 20.12, *p* = 0.28], 12 weeks (MD: 6.02, 95% CI: −0.22 to 12.26, *p* = 0.06), and in the long term at 24 weeks (MD: 2.25, 95% CI: −2.78 to 7.27, *p* = 0.38), as well as beyond 1 year (MD: 0.82, 95% CI: −1.75 to 3.39, *p* = 0.53). Compared to the cast immobilization group, the removable brace group showed similar rates of chronic regional pain [risk ratio (RR): 0.74, 95% CI: 0.14–3.94, *p* = 0.73], non-union (RR: 0.96, 95% CI: 0.17–5.46, *p* = 0.96), and thrombosis (RR: 0.46, 95% CI: 0.20–1.10, *p* = 0.08). Additionally, there was no significant difference in the incidence of wound infections when the brace was applied after primary wound healing (RR: 1.63, 95% CI: 0.87–3.03, *p* = 0.13). In terms of return to work (RTW), the removable brace group showed a significantly shorter mean time to return to work (MD: −17.17, 95% CI: −33.00 to −1.34, *p* = 0.03). Subgroup analysis revealed that the brace group achieved a better OMAS score at 12 weeks when early weight-bearing was permitted (MD: 9.00, 95% CI: 1.47–16.53, *p* = 0.02).

**Conclusion:**

Overall, both braces and casts demonstrated comparable effectiveness in postoperative ankle function recovery and wound complications. However, braces offered an advantage in promoting early weight-bearing, which contributed to improved recovery of ankle function. Additionally, the use of braces allowed patients to return to work earlier.

## Introduction

Ankle fractures are still one of the most common fracture types all over the world (1). Cast immobilization is a classic strategy for patients suffering ankle fractures, with apparent therapeutic effect and is being used widely (2). However, the disadvantages of cast immobilization have increasingly been brought to people’s attention, such as passive motion flexibility, reduction in strength, swelling, and pain (3, 4). According to reports, more patients have a preference for an alternative treatment, including removable brace (5, 6).

Removable braces, also known as functional orthoses or walking boots, are external devices that provide support to the ankle joint while allowing for rehabilitative movement during the recovery period. They typically consist of a rigid shell and adjustable straps, and may also include air cushions or rocker soles to facilitate walking (7). Compared to traditional casts, their removability helps reduce joint stiffness, improve early mobility, and facilitate a faster return to normal activities. Recent technological advancements have further enhanced the comfort, fit, and functional support of removable braces (8). As a result, they are increasingly used for stabilizing ankle fractures and in postoperative care, particularly for motivated patients who are able to adhere to rehabilitation protocols (9).

However, the controversy persists regarding whether a removable brace can serve as a substitute for cast immobilization after ankle fracture (9). Lehtonen et al. (8) concluded that the use of removable braces allows for greater joint freedom in the short term, but it also increases the risk of complications and cannot completely replace traditional cast immobilization. While Egol et al. (10) suggested that the removable braces enable patients to recover to their athletic ability sooner and achieve higher OMAS scores at 6 weeks and 12 weeks. In addition, there are two recent studies also supporting that a removable brace can serve as an alternative to cast immobilization (11–14).

Research on treatments for patients following ankle fractures remains an important topic. A meta-analysis (15) was conducted in 2023, with the conclusion that the wound complication rate of the removable brace group is 3 times that of the cast immobilization group. While the included studies were not comprehensive enough, the database search was incomplete, and there have been updates to related studies recently. To provide more comprehensive and up-to-date clinical evidence, we planned to conduct this meta-analysis. We hypothesize that the removable brace can serve as a viable alternative to cast immobilization. We will evaluate both treatments in terms of ankle joint functional scores, complications during the follow-up period, and time of return to work.

## Methods

This meta-analysis was performed according to the Preferred Reporting Items for Systematic Reviews and Meta-Analyses (PRISMA 2020) guidelines (16), and has been reported in line with the Assessment of Multiple Systematic Reviews-2 (AMSTAR 2) checklist and exhibits a high level of consistency with the AMSTAR 2 criteria (17). The study protocol has been registered on the International Prospective Register of Systematic Reviews (PROSPERO; CRD420251002317).

### Search strategy and selection criteria

A comprehensive literature search for studies comparing removable brace with cast immobilization after ankle fracture was conducted (last search on January 18, 2025) in the electronic databases including PubMed, The Cochrane Library, Embase, and Web of Science. A detailed search strategy was outlined in the Supplementary material.

The inclusion criteria included adults aged 18 years and older with an ankle fracture, no emergency circumstances, and a comparison of the removal of a brace and cast, with regular follow-up being feasible. The exclusion criteria included the following: Children below 18 years; a fracture secondary to known metastatic disease; complex intra-articular fracture; unstable fractures; full-text not available; and not in English. Meanwhile, the Population, Intervention, Comparison, Outcomes and Study (PICOS) criteria were utilized as a framework to structure the study question and develop the literature search strategies, ensuring comprehensive and unbiased searches:

P (Population): adults aged 18 years and older with an ankle fracture.

- I (Intervention): removable brace for treatment.
- C (Comparison): cast immobilization for treatment.
- O (Outcomes): functional ankle scores, complications, and RTW.
- S (Studies): prospective randomized controlled trials.

### Data extraction

Four reviewers independently extracted data from each included study using a standardized form, collecting information on study characteristics (first author, year of publication, and country), participant details (sample size, mean age with standard deviation, sex distribution, affected side, body mass index (BMI), and follow-up duration), and reported outcomes [functional scores such as OMAS and EuroQol-5D (EQ-5D), time to return to work, and major complications including thrombosis, regional pain, non-union, and wound infection]. To ensure consistency, all time-related data were standardized to weeks, and outcome measures were converted to standard scales when necessary. Discrepancies in data extraction or unit conversion were resolved through discussion or by consulting a third reviewer.

### Quality assessment

Three reviewers independently have assessed the included studies for the risk of bias using the Cochrane Collaboration’s risk of Bias Tool, which consists of seven domains of bias (random sequence generation, allocation concealment, blinding of participants and personnel, blinding of outcome assessment, incomplete outcome data, selective reporting, and other bias), in which each domain is divided into three levels, including low, unclear, or high risk of bias. Any discrepancies were resolved through discussion.

### Primary and secondary outcomes

The primary outcomes of this study were the differences in ankle function and health-related quality of life between the removable brace group and the cast immobilization group. Specifically, we assessed the Olerud–Molander Ankle Score (OMAS) at 6, 12, and 24 weeks, as well as beyond 1 year, and the EuroQol-5D (EQ-5D) score at 6 weeks. OMAS is a validated and widely used instrument for evaluating clinical outcomes in patients with ankle fractures, encompassing domains such as pain, stiffness, swelling, stair climbing, running, jumping, squatting, use of walking aids, and work/activity level (18). The total score ranges from 0 to 100, with higher scores indicating better ankle function. EQ-5D is a standardized measure of health-related quality of life that evaluates five dimensions: mobility, self-care, usual activities, pain/discomfort, and anxiety/depression. Higher scores reflect better perceived health status. Importantly, EQ-5D scores can be converted into utility values, enabling cost-effectiveness analyses of treatment interventions and providing valuable guidance for clinical decision-making and healthcare resource allocation (19).

According to previous studies, ankle function typically improves significantly within the first 3 months (12 weeks) after surgery, shows slight improvement between 3 and 6 months (24 weeks), and then tends to stabilize (20). Therefore, in this study, we extracted outcome data at 6, 12, and 24 weeks, and beyond 1 year to reflect postoperative ankle function recovery during the early phase (6 weeks), the period of marked improvement (12 weeks), the stabilization phase (24 weeks), and the long-term follow-up (more than 1 year).

Secondary outcomes included time to return to work, Visual Analog Scale (VAS) scores, and the incidence of major complications such as thromboembolism, complex regional pain syndrome (CRPS), non-union, and wound infection. Time to return to work, defined as the duration between surgery and the patient’s resumption of employment [PMID: 12571295; reference (8)], reflects the functional recovery of the ankle and the patient’s ability to resume daily and occupational activities. It is also critical for evaluating the socioeconomic impact of different treatment strategies (11, 20, 21). Moreover, postoperative complications such as thromboembolism (7–9, 11, 21, 22), CRPS (8, 11, 13, 22), non-union (8, 20, 22), and infection (8, 11, 21–23) are frequently observed following ankle fractures due to limb immobilization, cast or brace compression, and reduced weight-bearing. Monitoring the incidence of these adverse events is essential for supporting optimal postoperative recovery. In summary, assessing these secondary outcomes provides valuable insights into the safety and efficacy of removable braces in the management of ankle fractures.

### Statistical analysis

Dichotomous variables, including complications such as thromboembolism, complex regional pain syndrome (CRPS), non-union, and wound infection, were extracted and presented as the number of events and total cases. Continuous variables, including functional scores (OMAS and EQ-5D), time to return to work, and pain intensity, were summarized using means and standard deviations. The results for binary variables were depicted using risk ratios (RR) along with 95% confidence intervals (CIs) to convey pooled outcomes. Additionally, outcomes for continuous variables were presented using mean differences (MD) and 95% confidence intervals for pooled results. The meta-analysis employed the inverse-variance method to assign weights to each study. This approach calculates study weights based on the inverse of the variance of the effect estimate, such that studies with smaller variances (i.e., more precise estimates) are given greater weight in the overall pooled effect. Compared with weighting solely by sample size or event frequency, inverse–variance weighting better captures the reliability and informational contribution of each study, enhancing the accuracy and robustness of the pooled results. Considering the specific conditions of fractures, the types of orthopedic supports, and potential heterogeneity due to variations in early weight-bearing across studies, we employed a random-effects model. Subgroup analysis was also carried out based on early weight-bearing implementation, brace characteristics, and patient characteristics. Sensitivity analysis was conducted on the results using the leave-one-out method to assess the robustness of the findings. This sensitivity analysis was planned *a priori* as part of our study design to determine whether any individual study disproportionately influenced the pooled effect estimates, ensuring that the conclusions are not driven by outliers or studies with potential bias. All analyses were performed using Review Manager 5.4 (RevMan 5.4), The Cochrane Collaboration.

## Results

### Literature search

**Figure 1:**
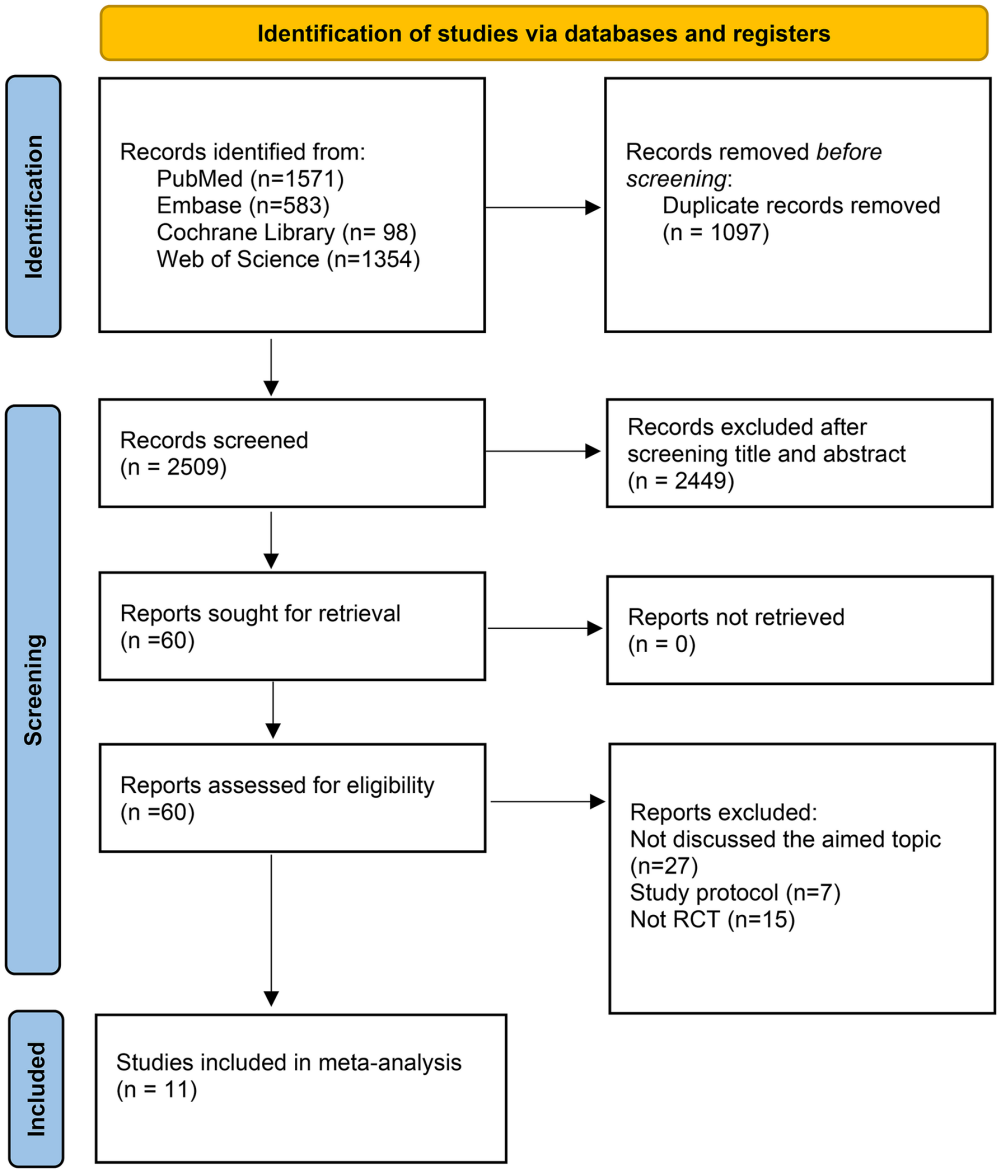
Search flowchart.

PubMed, Cochrane Library, Embase, and Web of Science were last searched on June 27, 2025, to gather relevant literature on this topic. Our initial search yielded 3,606 articles, from which 1,097 duplicates were removed. Following a preliminary screening of titles and abstracts, an additional 2,449 articles were excluded. After a careful review of the full texts, 49 articles that did not meet our eligibility criteria were further omitted. At last, our search resulted in the inclusion of 11 randomized controlled trials (RCTs) (8, 11, 12, 21–28). The search flowchart is shown in Figure 1.

### Baseline study characteristics

We included 11 RCTs and 1,472 participants, with the mean age being 44.4 years. The two groups were similar in terms of patient demographics and fracture characteristics. The mean age, male/female ratio, left/right ankle fracture distribution, and mean BMI were comparable between the removable brace group and the cast immobilization group. There was a variation in the duration of brace and cast wearing among different studies, with 3 weeks of treatment in one study (11), 4 weeks of treatment in one study (23), and 6 weeks of treatment in 8 studies (8, 12, 21, 22, 25–28), while one study (24) compared brace wearing for 3 weeks with cast wearing for 6 weeks. Finally, the duration of follow-up varied from 12 weeks to 2 years. The baseline demographics and injury-related study characteristics are shown in Table 1. The characteristics of the removable brace in each study are presented in Supplementary Tables 1, 2.

**Table 1:** Baseline demographics and injury-related characteristics of included RCTs.

Study	Location	Sample size, *n* (%)		Age, mean (SD), years		Sex (male/female), *n* (%)		Side (left/right), *n* (%)		Follow-up time
		Brace	Cast	Brace	Cast	Brace	Cast	Brace	Cast	
Kearney et al. (11)	UK	335 (50%)	334 (50%)	45.9 (16)	46.9 (17.0)	152 (45%)/183 (55%)	136 (41%)/198 (59%)	161 (49%)/170 (51%)	176 (53%)/158 (47%)	16 weeks
Bayram et al. (23)	Turkey	33 (51%)	32 (49%)	47.0 (13.1)	45.4 (13.1)	10 (30%)/23 (70%)	9 (28%)/23 (72%)	22 (67%)/11 (33%)	20 (63%)/12 (37%)	24 weeks
Lehtonen et al. (8)	Finland	50 (50%)	50 (50%)	41.0 (13.0)	41.0 (13.0)	25 (50%)/25 (50%)	31 (62%)/19 (38%)	22 (44%)/28 (56%)	20 (40%)/30 (60%)	2 years
Egol et al. (10)	USA	27 (49%)	28 (50%)	39.5 (17.2)	45.6 (17.5)	13 (48%)/14 (52%)	10 (36%)/18 (64%)	8 (30%)/19 (70%)	12 (43%)/16 (57%)	1 year
Kearney et al. (21)	UK	25 (50%)	25 (50%)	40.7 (17.1)	42.7 (14.9)	10 (40%)/15 (60%)	14 (56%)/11 (44%)	10 (40%)/15 (60%)	11 (44%)/14 (56%)	2 years
Stassen et al. (12)	The Netherlands	25 (51%)	24 (49%)	52 (19.0)	51.0 (15.0)	17 (68%)/8 (32%)	7 (29%)/17 (71%)	NA	NA	12 weeks
van den Berg et al. (25)	The Netherlands	23 (52%)	21 (48%)	44.3 (14.9)	41.9 (16.3)	13 (57%)/10 (43%)	11 (52%)/10 (48%)	NA	NA	1 year
Kortekangas et al. (24)	Finland	80 (49%)	84 (51%)	46.0 (19.0)	46.0 (17.0)	36 (45%)/44 (55%)	46 (55%)/38 (45%)	NA	NA	1 year
Dehghan et al. (22)	Canada	56 (51%)	54 (49%)	41.7 (15.1)	42.1 (15.4)	32 (57%)/24 (43%)	27 (50%)/27 (50%)	28 (50%)/28 (50%)	23 (43%)/31 (57%)	1 year
Jarragh et al. (27)	Kuwait	54 (52%)	50 (48%)	40 (11.1)	39.5 (13.0)	33 (61%)/21 (39%)	36 (72%)/14 (28%)	26 (48%)/28 (52%)	22 (44%)/28 (56%)	1 year
Vioreanu et al. (28)	Ireland	33 (53%)	29 (47%)	37.2 (12.9)	34.9 (16)	21 (68%)/10 (32%)	20 (69%)/9 (31%)	NA	NA	12 weeks

NA, not available.

### Quality assessment

**Figure 2:**
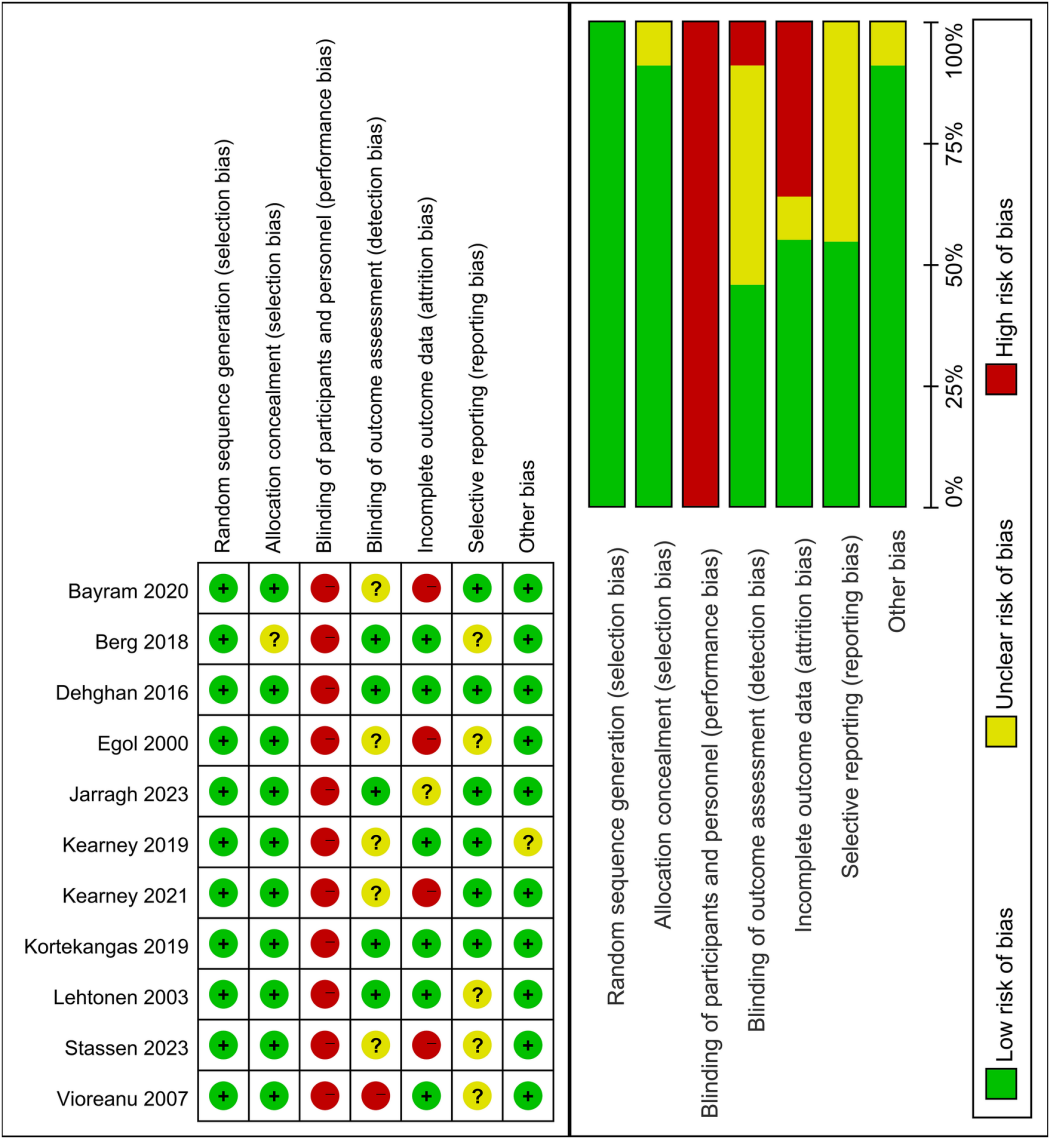
Distribution of risk of bias of included RCTs.

All studies (8, 10–12, 21–28) were rated as having a high risk in terms of blinding of participants and personnel (performance bias) because the operation between removable brace and cast immobilization was unlikely to adopt blinding due to the nature of the interventions. Then, the incomplete outcome data occurred in four studies (10–12, 23) included, resulting in a deficit rate of more than 20% during the follow-up period. However, these should not cause bias in the analysis, given that the outcomes were objectively assessed and the blinding of outcome assessment was well implemented. There were many unclear biases in the blinding of outcome assessment and report selection, mainly due to the lack of a detailed description of the outcome collection and evaluation, and the incomplete reporting of results. The distribution of the bias has been shown in Figure 2.

### Functional ankle scores

**Figure 3:**
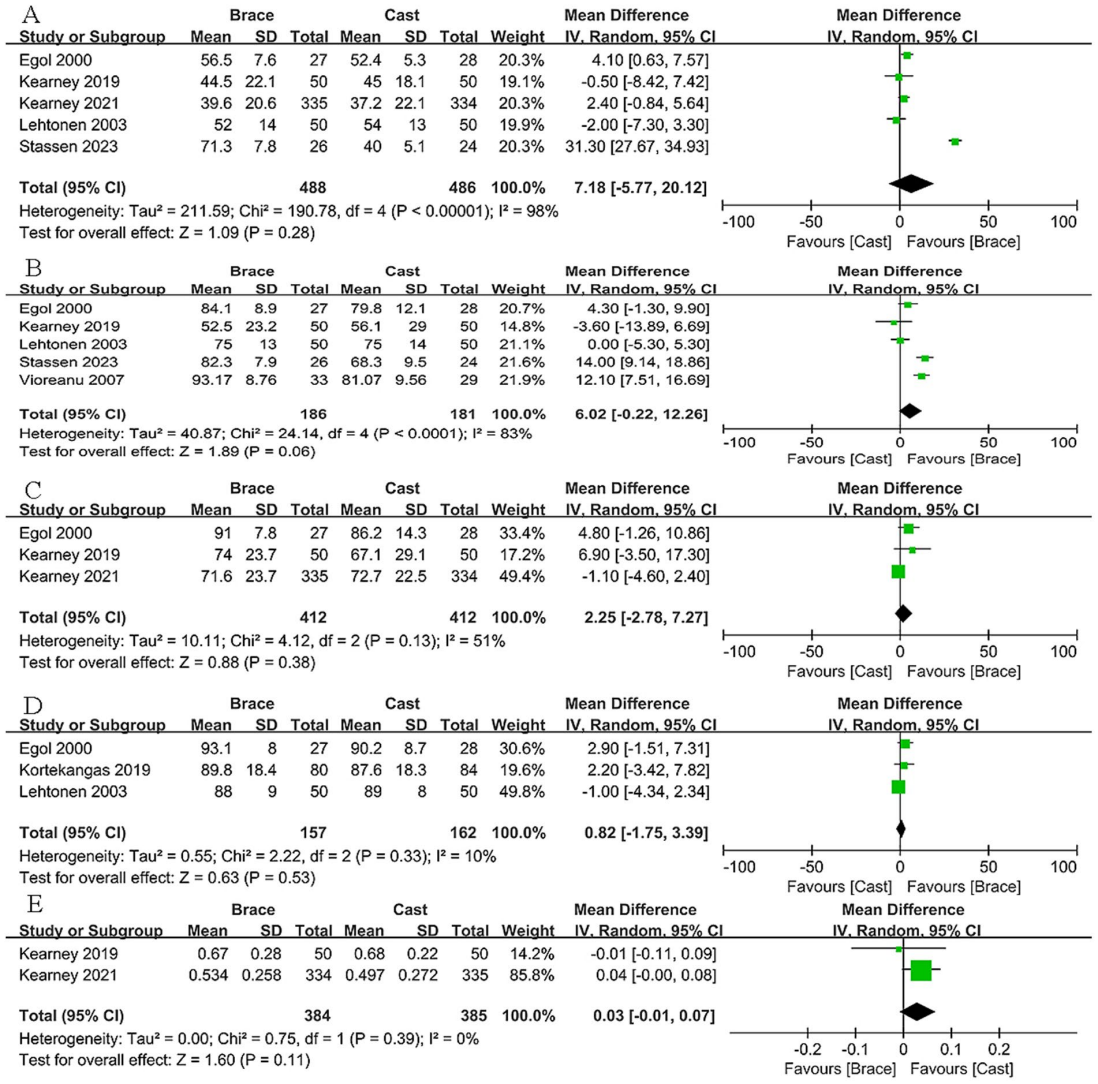
OMAS scores at 6 weeks **(A)**, 12 weeks **(B)**, 24 weeks **(C)**, and 1 year **(D)**, and EQ-5D scores at 6 weeks **(E)**.

The major OMAS were reported in seven studies included. During the short-term follow-up period, there are no significant differences of OMAS between removable brace and cast immobilization at 6 weeks (MD: 7.18, 95% CI: −5.77 to 20.12, *p* = 0.28; *I*^2^ = 98%) (Figure 3A), 12 weeks (MD: 6.02, 95% CI: −0.22 to 12.26, *p* = 0.06; *I*^2^ = 83%) (Figure 3B), and during the follow-up period of long-term, no significant statistical differences were found in OMAS scores comparison either at 24 weeks (MD: 2.25, 95% CI: −2.78 to 7.27, *p* = 0.38; *I*^2^ = 51%) (Figure 3C) or at even more than 1 year (MD: 0.82, 95% CI: −1.75 to 3.39, *p* = 0.53; *I*^2^ = 10%) (Figure 3D).

In addition, the EuroQol-5D scores were reported in two studies included (11, 21). There was also no significant discrepancy between the two groups in the short-term period, as EuroQol-5D scores at 6 weeks (MD: 0.03, 95% CI: −0.01 to 0.07, *p* = 0.11; *I*^2^ = 0%) (Figure 3E).

### Complications

The complications related to treatment methods were reported in nine studies included (8, 11, 12, 22–28). We have selected four main complications that were commonly mentioned in these studies.

**Figure 4:**
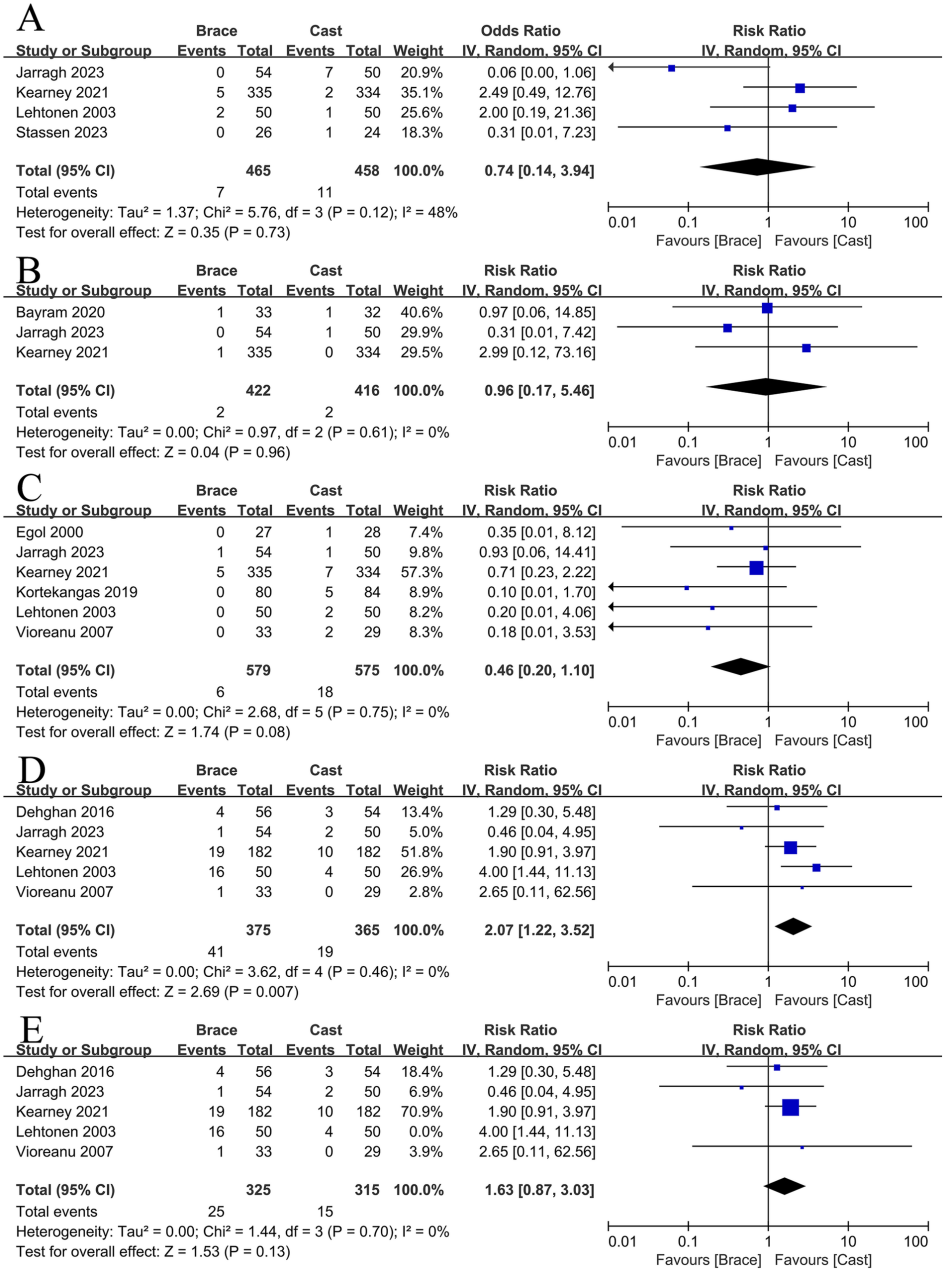
The risk of developing chronic regional pain **(A)**, the incidence of non-union **(B)**, the risk of developing thrombosis **(C)**, the incidence of wound infections **(D)**, and the incidence of wound infections after excluding studies with high risk of bias **(E)**.

The removable brace group is similar in terms of developing chronic regional pain (RR: 0.74, 95% CI: 0.14–3.94, *p* = 0.73; *I*^2^ = 48%) (Figure 4A) and non-union (RR: 0.96, 95% CI: 0.17–5.46, *p* = 0.96; *I*^2^ = 0%) (Figure 4B), compared with cast immobilization group. In addition, the removable brace group is less likely to form thrombus even without sufficient statistical significance (RR: 0.46, 95% CI: 0.20–1.10, *p* = 0.08; *I*^2^ = 0%) (Figure 4C). However, a removable brace has a higher incidence of wound infections (RR: 2.07, 95% CI: 1.22–3.52, *p* = 0.007; *I*^2^ = 0%) (Figure 4D). To explore the possible reasons for the higher wound infection rates in the brace group, we excluded a study (8) with significant controversy for the brace being applied immediately after surgery, while the remaining four studies (11, 22, 27, 28) mentioned wearing the brace after the primary wound healing. Based on these four studies, there was no significant difference between the two groups in terms of wound infections (RR: 1.63, 95% CI: 0.87–3.03, *p* = 0.13; *I*^2^ = 92%) (Figure 4E).

### Return to work

RTW (return to work) was reported in detail in the four studies (8, 10, 23, 28) included. The mean time of RTW of the removable brace group is shorter than that of the cast immobilization group, and there is a significant difference between the two groups (Favours Brace, MD: −17.17, 95% CI: −33.00 to −1.34, *p* = 0.03; *I*^2^ = 92%) (Supplementary Figure 1).

### Subgroup analysis

Four studies (8, 10, 27, 28) included explicitly specified that weight-bearing was not allowed until 6 weeks postoperatively, whereas other studies allowed early weight-bearing as soon as possible. To investigate the impact of early weight-bearing on ankle joint functional scores, we conducted a subgroup analysis based on weight-bearing before or after 6 weeks. When weight-bearing commenced within 6 weeks postoperatively, there was no statistically significant difference in the 6-week OMAS scores between the brace and cast (MD: 11.19, 95% CI: −10.51 to 32.90, *p* = 0.31; *I*^2^ = 99%) (Supplementary Figure 2A). However, the 12-week OMAS scores for the brace were significantly higher than for the cast (Favours Brace, MD: 9.00, 95% CI: 1.47–16.53, *p* = 0.02; *I*^2^ = 79%) (Supplementary Figure 2B). When weight-bearing commenced after 6 weeks postoperatively, there was no significant difference in 6-week OMAS scores (MD: 1.39, 95% CI: −4.55 to 7.33, *p* = 0.65; *I*^2^ = 72%) (Supplementary Figure 2C) and 12-week OMAS scores (MD: 2.05, 95% CI: −2.16 to 6.26, *p* = 0.34; *I*^2^ = 16%) (Supplementary Figure 2D) between the brace and cast.

Furthermore, considering that surgical reduction has a significant impact on the results, we conducted a subgroup analysis in terms of functional scores to determine whether surgical treatment was performed before immobilization of ankle fractures. Six studies (7, 9, 16, 17, 22, 23) included all patients being treated surgically, and four studies (10, 15, 18, 19) included all patients being treated non-surgically. We have made a comparison and analysis in the surgical group between the two immobilization methods at different follow-up periods. Consequently, except for the higher OMAS scores with removable brace at 24 weeks (MD: 5.33, 95% CI: 0.10–10.57, *p* = 0.05; *I*^2^ = 50%) (Supplementary Figure 3C), no other significant differences were found at 6 weeks (MD: 1.14, 95% CI: −3.14 to 5.43, *p* = 0.60; *I*^2^ = 80%) (Supplementary Figure 3A), at 12 weeks (MD: 3.88, 95% CI: −2.87 to 12.62, *p* = 0.26; *I*^2^ = 0%) (Supplementary Figure 3B), and at 1 year (MD: 0.67, 95% CI: −3.11 to 4.45, *p* = 0.73; *I*^2^ = 48%) (Supplementary Figure 3D). As for the nonoperative group, we selected OMAS scores and pain of VAS in the short term to evaluate and compare the two immobilization methods. There were no significant differences with OMAS scores at 6 weeks (MD: 13.26, 95% CI: −22.32 to 48.83, *p* = 0.47; *I*^2^ = 99%) (Supplementary Figure 4A), at 12 weeks (MD: 7.79, 95% CI: −4.94 to 20.51, *p* = 0.23; *I*^2^ = 88%) (Supplementary Figure 4B), and with VAS at 6 weeks (MD: 0.11, 95% CI: −2.03 to 2.26, *p* = 0.92; *I*^2^ = 85%) (Supplementary Figure 4C) and at 12 weeks (MD: 0.15, 95% CI: −0.51 to 0.81, *p* = 0.65; *I*^2^ = 0%) (Supplementary Figure 4D).

At last, to minimize the impact of different types and brands of removable braces on the results, we conducted a subgroup analysis regarding OMAS scores based on whether the brace angle is adjustable, with five studies in the non-adjustable group and five studies in the adjustable group. No significant differences were found in the non-adjustable group at 6 weeks (MD: −0.36, 95% CI: −5.02 to 4.30, *p* = 0.88; *I*^2^ = 51%) (Supplementary Figure 5A) and at 12 weeks (MD: 3.90, 95% CI: −5.91 to 13.71, *p* = 0.44; *I*^2^ = 83%) (Supplementary Figure 5B) or in the adjustable group at 6 weeks (MD: 11.18, 95% CI: −9.23 to 31.95, *p* = 0.28; *I*^2^ = 99%) (Supplementary Figure 5C) and at 12 weeks (MD: 6.16, 95% CI: −2.27 to 14.60, *p* = 0.15; *I*^2^ = 87%) (Supplementary Figure 5D).

### Sensitivity analysis

A sensitivity analysis was performed on the aforementioned results using the leave-one-out method. Robustness was maintained in all outcomes, except for the incidence of wound infection. After excluding the studies by Kearney et al. and Lehtonen et al., respectively, no difference in wound infection was observed between the brace and cast groups.

## Discussion

Cast immobilization remains a conventional method for treating ankle fractures due to its stability and safety. However, with increasing awareness of its drawbacks—such as discomfort, muscle atrophy, and loss of strength—a growing number of clinical trials have compared casts with removable braces in recent years (11, 20, 22, 28). These studies generally support the comparable effectiveness of the two approaches in terms of functional recovery and complication control. A previous meta-analysis by Li et al. (15) reported that the incidence of wound complications was three times higher in the brace group compared to the cast group. However, that study included only five RCTs and did not conduct subgroup analyses; notably, one trial (11) reported an unusually high infection rate in the brace group (66% vs. 16%, *p* = 0.0005) due to inappropriate brace application timing. To address these limitations, the present meta-analysis included 11 RCTs and, for the first time, simultaneously evaluated efficacy, safety, and cost, aiming to provide a more comprehensive comparison.

Our results showed no significant difference in overall functional outcomes between braces and casts. Notably, early weight-bearing led to higher OMAS scores at 12 weeks in the brace group, consistent with prior findings (26). Moreover, several studies reported better dorsiflexion, plantarflexion, and physical and mental scores of the 36-item Short Form Health Survey (SF-36) in the early posttreatment phase for the brace group (9, 11, 13, 20, 21, 23, 24).

However, high heterogeneity was observed in several key outcomes, such as functional scores at 6 weeks (*I*^2^ = 98%) and 12 weeks (*I*^2^ = 83%), as well as trauma infection rates (*I*^2^ = 92%). This variability likely stems from differences in treatment protocols (e.g., immobilization duration and timing of weight-bearing), inclusion of both surgical and non-surgical patients, and patient baseline characteristics (e.g., age, BMI, and fracture type). To further explore heterogeneity, we conducted subgroup analyses based on time points (e.g., 6 weeks and 12 weeks), brace type, weight-bearing timing (early vs. delayed), and surgical vs. non-surgical management. While these analyses provided useful insights—such as the importance of early mobilization—they should be interpreted cautiously. The included studies varied in protocols, interventions, and participant characteristics, which may introduce confounding factors and limit the reliability of direct comparisons. Therefore, these findings are exploratory rather than confirmatory. Future research should adopt standardized treatment approaches and more homogeneous participant criteria, along with longer follow-up durations, to validate these findings and better understand the effects of early mobilization and other protocol variations on long-term outcomes.

We also conducted a leave-one-out sensitivity analysis, which showed that the study by Stassen (12) had a significant impact on the primary outcome—OMAS score. Although the sample size of this study was relatively small (26 participants in the treatment group and 24 in the control group), it reported a markedly higher mean difference (31.3, 95% CI: 27.67–34.93), likely due to its small standard deviations and consequently higher statistical weight (20.3%). After excluding this study, the pooled mean difference decreased to 1.9 (95% CI: −0.64 to 4.44), suggesting that this study partially contributed to the overall trend. Therefore, caution should be exercised when interpreting the OMAS results, although OMAS remains a standardized and valid tool for assessing ankle joint function.

In terms of complications, we found no significant group differences in the incidence of CRPS or non-union. The cast group showed a trend toward higher thrombosis rates, although this was not statistically significant. A higher infection rate in the brace group was likely attributable to the early application before wound healing had occurred. When braces were applied after initial wound healing, infection rates were comparable, suggesting that appropriate timing mitigates this risk.

Furthermore, our analysis demonstrated that patients in the brace group returned to work significantly earlier than those in the cast group. This finding aligns with previous results, which show that early mobilization shortens return-to-work time (91.3 ± 20.2 vs. 54.6 ± 15.5 days), potentially reducing indirect costs (30).

Ankle fractures incur both direct and indirect healthcare costs. Jarragh et al. (27) reported a significantly higher emergency department visit rate in the plaster splint group compared to the non-splint group (46% vs. 7.4%, *p* < 0.001), primarily due to plaster-related discomfort, stiffness, or complications such as compartment syndrome. These repeated visits contribute to increased direct medical costs. Despite regional differences in clinical practice, existing studies suggest that the overall cost of removable braces is comparable to or even lower than that of traditional casting (9, 29, 30). Additionally, a cost-utility analysis by Nwankwo et al. (31) showed that using a removable brace for ankle fractures resulted in an incremental cost of £46.73 (95% CI: £-9–£147), with a cost per quality-adjusted life year (QALY) gained of £3,318. The probability of the brace being cost-effective at a £30,000/QALY willingness-to-pay threshold was 88%. These findings align with our conclusion, supporting the potential economic benefit of removable braces. However, considering the heterogeneity in treatment protocols and patient populations across studies, the cost-effectiveness results should be interpreted cautiously, and future research is needed to validate these findings in different healthcare systems.

Patient comfort and satisfaction are important, but were inconsistently reported, preventing their inclusion in the meta-analysis. One study (Jarragh et al.) (8) showed higher satisfaction with braces at 3 and 6 weeks, suggesting better short-term comfort. However, by 12 months, satisfaction levels between brace and cast groups were similar, indicating minimal long-term differences. These findings imply that braces may offer early patient preference, but long-term satisfaction converges. Further research is needed on long-term patient-reported outcomes.

It is important to acknowledge that variability in brace designs and treatment protocols among the included studies may have introduced bias and contributed to the observed heterogeneity. Some studies used fixed-angle, non-adjustable braces [e.g., (11, 24)], while others employed adjustable designs [e.g., (8, 10, 23)]. Additionally, in multicenter studies like Kearney et al. (11), different sites used different brace brands, further increasing variability. This lack of standardization limits the generalizability of the findings and may affect treatment outcomes. There were also notable differences in intervention protocols, such as weight-bearing timing (early vs. delayed) and immobilization duration (ranging from 3 to 6 weeks), which may have influenced functional recovery and complication rates. Although subgroup analyses were performed to address these factors, residual heterogeneity remains. Therefore, future studies should aim to standardize brace types and treatment protocols to reduce bias and improve comparability. Overall, while both removable braces and cast immobilization appear effective, consistent methodology in future trials is essential for drawing more robust conclusions regarding their relative benefits for ankle fracture management.

Compared with the meta-analysis conducted by Zhou et al. (29), which included 11 studies—approximately 30% of which were retrospective in design and exhibited notable heterogeneity—the present study incorporated only randomized controlled trials to ensure higher methodological quality. While Zhou et al. used a fixed-effects model, which may overestimate treatment effects in the presence of heterogeneity, we adopted a random-effects model to yield more conservative and reliable estimates. Moreover, our analysis not only focused on functional outcomes and complications but also included additional clinically relevant outcomes such as time to weight-bearing and return to work. As a result, although both studies observed comparable functional recovery between braces and casts, our findings suggest that braces may offer advantages in promoting earlier weight-bearing and facilitating an earlier return to work, thus providing a valuable alternative in the clinical management of ankle fractures.

However, this study has several limitations. First, the number of included studies and patients remains relatively small, and there was considerable heterogeneity among trials, which may affect the robustness of the conclusions. Second, regarding the analysis of complications, due to insufficient data to support a broader evaluation, we only analyzed the most commonly reported complications, which resulted in less comprehensive findings. Third, although some studies reported the affected side (left or right) as part of baseline characteristics, none conducted subgroup analyses based on limb dominance. As a result, we were unable to evaluate whether dominance influenced functional outcomes or introduced additional heterogeneity across studies. Fourth, although all included studies employed removable braces as an intervention, significant differences existed in brace type, adjustability, and duration of use, as shown in Table 1. Additionally, variations in brace brands and specific application methods may have introduced a certain degree of clinical heterogeneity. However, since all braces share the core function of providing ankle joint stability and facilitating functional recovery, we believe their fundamental therapeutic intent is consistent, and these differences are unlikely to have a substantial impact on the primary outcomes. Lastly, due to the limited number of studies available for each specific type of brace, we were unable to conduct subgroup analyses based on brace type. Therefore, our findings primarily reflect an overall comparison between removable braces and cast immobilization and may not be generalizable to every specific type of removable brace.

## Conclusion

There are no statistically significant differences between removable braces and cast immobilization in terms of OMAS scores and EQ-5D scores, both in the short-term and long-term periods. Regarding complications, the brace is non-inferior to the cast. In terms of return to work, the brace group facilitates a quicker return to work compared to the cast group. Meanwhile, early weight-bearing with braces can contribute to better recovery of ankle function for patients. Therefore, removable braces can be considered a viable alternative to cast immobilization for ankle fractures.

## Data Availability

The original contributions presented in the study are included in the article/Supplementary material, further inquiries can be directed to the corresponding authors.
