# Supplementary material for: Removable brace as a viable alternative to cast immobilization for ankle fractures—a meta-analysis of randomized controlled trials
Source: Front Med (Lausanne). 2025 Jul 29;12:1594505. doi: 10.3389/fmed.2025.1594505 (PMC12339471; doi:10.3389/fmed.2025.1594505)

**Search strategy** Page2-4

**eTable1. Characteristic of removable brace of each study**  Page5

**eTable2. Summary of Patient Characteristics and Intervention Details in Included Studies** Page6-9

**eFigure1. The mean time of RTW between Removable brace and Cast immobilization.** Page10

**eFigure2. Subgroup analysis: Early WB OMAS scores at 6 weeks (A),12 weeks (B); Late WB OMAS scores at 6 weeks (C),12 weeks (D).** Page11

**eFigure3. Subgroup analysis: Operative patients’ OMAS scores at 6 weeks (A), 12 weeks (B), 24 weeks (C), 1 year (D).** Page12

**eFigure4. Subgroup analysis: Nonoperative patients’ OMAS scores at 6 weeks (A), 12 weeks (B) and VAS scores at 6 weeks (C), 12 weeks (D).** Page13

**eFigure5. Subgroup analysis: Non-adjustable group’s OMAS scores at 6 weeks (A), 12 weeks (B) and Adjustable group’s OMAS scores at 6 weeks (C), 12 weeks (D).** Page14

**PubMed**

1. “Ankle Fracture” [Mesh] OR “Fracture, Ankle” [Mesh] OR “Ankle Fracture” [tiab]

2. “Bimalleolar Fractures” [tiab] OR “Bimalleolar Fracture”[tiab] OR “Fracture,Bimalleolar” [tiab] OR “Bimalleolar Ankle Fractures”[tiab] OR “Ankle Fracture, Bimalleolar”[tiab] OR “Bimalleolar Ankle Fracture”[tiab] OR “Fracture, Bimalleolar Ankle”[tiab] OR “Lateral Malleolus Fractures”[tiab] OR “Fracture, Lateral Malleolus”[tiab] OR “Lateral Malleolus Fracture”[tiab] OR “Malleolus Fracture, Lateral”[tiab] OR “Trimalleolar Fractures”[tiab] OR “Fracture, Trimalleolar”[tiab] OR “Trimalleolar Fracture”[tiab] OR “Trimalleolar Ankle Fractures”[tiab] OR “Ankle Fracture, Trimalleolar”[tiab] OR“Fracture, Trimalleolar Ankle”[tiab] OR“Trimalleolar Ankle Fracture”[tiab] OR “Medial Malleolus Fractures”[tiab] OR “Fracture, Medial Malleolus”[tiab] OR “Malleolus Fracture, Medial”[tiab] OR “Medial Malleolus Fracture”[tiab] OR “Posterior Malleolus Fractures” [tiab] OR “Fracture, Posterior Malleolus” [tiab] OR “Malleolus Fracture, Posterior” [tiab] OR “Posterior Malleolus Fracture” [tiab] OR “Bimalleolar Equivalent Fractures” [tiab] OR “Bimalleolar Equivalent Fracture” [tiab] OR “Equivalent Fracture, Bimalleolar” [tiab] OR “Fracture, Bimalleolar Equivalent” [tiab] OR “Bimalleolar Equivalent Ankle Fractures” [tiab] OR “foot fracture”[tiab]

3. #1 OR #2

4. “Surgical Casts”[tiab] or “Cast, Surgical” [tiab] or “Surgical Cast” [tiab] or “Plastic Casts” [tiab] or “Cast, Plastic” [tiab] or “Casts, Plastic” [tiab] or “Plastic Cast” [tiab] or “Plaster Casts” [tiab] or “Cast, Plaster” [tiab] or “Casts, Plaster” [tiab] or “Plaster Cast” [tiab] or “Fiberglass Casts” [tiab] or “Cast, Fiberglass” [tiab] or “Casts, Fiberglass” [tiab] or “Fiberglass Cast” [tiab]

5. “brace”[Mesh] or “brace”[tiab]

6. #4 OR #5

7. (compar*[tiab]) OR ((singl*[tiab] or doubl*[tiab] or tripl*[tiab]) and (mask*[tiab] or blind*[tiab])) )OR (random*[tiab] or placebo[tiab] or controlled[tiab] or trial*[tiab] #3 AND #6 AND #7

**Cochrane Library**

1. (Bimalleolar Ankle Fracture OR Bimalleolar Ankle Fractures OR Fracture, Bimalleolar Ankle OR Ankle Fracture, Bimalleolar OR Bimalleolar Fractures OR Bimalleolar Fracture OR Fracture, Bimalleolar OR Malleolus Fracture, Medial OR Medial Malleolus Fractures OR Fracture, Medial Malleolus OR Medial Malleolus Fracture OR Lateral Malleolus Fracture OR Lateral Malleolus Fractures OR Fracture, Lateral Malleolus OR Malleolus Fracture, Lateral OR Ankle Fracture OR Fracture, Ankle OR Trimalleolar Ankle Fracture OR Fracture, Trimalleolar OR Trimalleolar Fractures OR Fracture, Trimalleolar Ankle OR Trimalleolar Ankle Fractures OR Trimalleolar Fracture OR Ankle Fracture, Trimalleolar OR Posterior Malleolus Fractures OR Malleolus Fracture, Posterior OR Fracture, Posterior Malleolus OR Posterior Malleolus Fracture OR Bimalleolar Equivalent Ankle Fractures OR Bimalleolar Equivalent Fracture OR Bimalleolar Equivalent Fractures OR Fracture, Bimalleolar Equivalent OR Equivalent Fracture, Bimalleolar ):ti,ab

2. (Casts, Plastic OR Plastic Casts OR Plastic Cast OR Cast, Plastic OR Surgical Cast OR Cast, Surgical OR Surgical Casts OR Fiberglass Casts OR Casts, Fiberglass OR Fiberglass Cast OR Cast, Fiberglass OR Casts, Plaster OR Plaster Cast OR Cast, Plaster OR Plaster Casts ):ti,ab

3. (Brace ):ti,ab

4. ((compar*) OR ((singl* or doubl* or tripl*) and (mask* or blind*))) OR (random* or placebo or controlled or trial*):ti,ab

5. MeSH descriptor: [Ankle Fractures] explode all trees

6. MeSH descriptor: [Casts, Surgical] explode all trees

7. MeSH descriptor: [Braces] explode all trees

8. (#1 OR #5) AND (#2 OR #3 OR #6 OR #7) AND #4

**Embase**

1. ‘ankle fractures’/exp OR ( (ankle fracture malunion) OR (ankle fractures) OR (ankle syndesmosis rupture) OR (broken ankle) OR (fracture, ankle) OR (fractured ankle) OR (ankle fracture) ):ti,ab

2. ‘cast’/exp OR ( (cast, plaster) OR (plaster bandage) OR (plaster dressing) OR (plaster splint) OR (plaster-of-Paris cast) OR (Polyform (plaster cast)) OR (Specialist (plaster cast)) OR (splint, plaster) OR (plaster cast) ):ti,ab

3. ‘brace’/exp OR ( (braces) OR (braces-orthopaedic appliances) OR (braces-orthopedic appliances) OR (bracing) OR (orthopaedic brace) OR (orthopaedic braces) OR (orthopedic brace) OR (orthopedic braces) OR (X-Act (brace)) OR (brace) ):ti,ab

4. #2 OR #3

5. ((compar*) OR ((singl* or doubl* or tripl*) and (mask* or blind*))) OR (random* or placebo or controlled or trial*):ti,ab

6. #1 AND #4 AND #5

**Web of Science**

1. TS=(Ankle Fracture OR Fracture, Ankle OR Ankle Fracture OR Bimalleolar Fractures Bimalleolar Bimalleolar Ankle Fractures Fracture OR OR Fracture,Bimalleolar OR Ankle Fracture, Bimalleolar OR Bimalleolar Ankle Fracture OR Fracture, Bimalleolar Ankle OR Lateral Malleolus Fractures OR Fracture, Lateral Malleolus OR Lateral Malleolus Fracture OR Malleolus Fracture, Lateral OR Trimalleolar Fractures OR Fracture, Trimalleolar OR Trimalleolar Fracture OR Trimalleolar Ankle Fractures OR Ankle Fracture, Trimalleolar ORFracture, Trimalleolar Ankle ORTrimalleolar Ankle Fracture OR Medial Malleolus Fractures OR Fracture, Medial Malleolus OR Malleolus Fracture, Medial OR Medial Malleolus Fracture OR Posterior Malleolus Fractures OR Fracture, Posterior Malleolus OR Malleolus Fracture, Posterior OR Posterior Malleolus Fracture OR Bimalleolar Equivalent Fractures OR Bimalleolar Equivalent Fracture OR Equivalent Fracture, Bimalleolar OR Fracture, Bimalleolar Equivalent OR Bimalleolar Equivalent Ankle Fractures OR foot fracture) and Preprint Citation Index

2. TS=(Surgical Casts or Cast, Surgical or Surgical Cast or Plastic Casts or Cast,Plastic or Casts, Plastic or Plastic Cast or Plaster Casts or Cast, Plaster or Casts, Plaster or Plaster Cast or Fiberglass Casts or Cast, Fiberglass or Casts, Fiberglass or Fiberglass Cast) and Preprint Citation Index

3. TS=(brace ) and Preprint Citation Index

4. #3 OR #2

5. TS=(((compar*) OR ((singl* or doubl* or tripl*) and (mask* or blind*))) OR (random* or placebo or controlled or trial*)) and Preprint Citation Index

6. #1 AND #5 AND #4

eTable1. Characteristic of removable brace of each study

| Study | Description of removable brace in study | The adjustability of the angle |
| --- | --- | --- |
| Kearney 2021 | Removable braces were of a  fixed angle design, to replicate  what is routinely used in UK  practice, and were applied in  accordance with local  procedures. The specific brand  of removable brace was not  standardised across sites, and  each site used its own brand. | Non-adjustable |
| Bayram 2020 | Double-layered elasticated bandage | Adjustable |
| Lehtonen 2003 | Functional ankle brace | Adjustable |
| Egol 2000 | Removable functional brace (Aircast, Summit, New Jersey) | Adjustable |
| Kearney 2019 | Fixed angle removable orthotic | Non-adjustable |
| Stassen 2023 | Walking boot (Rebound® Air Walker, Össur, Reykjavik, Iceland) | Adjustable |
| Berg 2018 | Removable ankle brace (Bauerfeind Malleoloc) | Unknown |
| Kortekangas 2019 | Simple orthotic device (Dynacast/Ortho-Glass; BSN medical, Rutherford College, NC) | Non-adjustable |
| Dehghan 2016 | Boot orthosis | Non-adjustable |
| Jarragh 2023 | Removable, functional ankle brace | Adjustable |
| Vioreanu 2007 | Custom-made removable fiberglass cast | Non-adjustable |

eTable2. Summary of Patient Characteristics and Intervention Details in Included Studies

| Study | Inclusion Criteria | BMI (Brace) | BMI (Cast) | Brace Intervention | Cast Intervention |
| --- | --- | --- | --- | --- | --- |
| Kearney 2021 | Adults ≥18 years with closed ankle fractures, regardless of surgical treatment | 28.6 | 28.2 | Removable fixed-angle brace (brand varied by center) applied per local protocol. Participants were verbally and in writing encouraged to remove the brace frequently for non-weight-bearing ankle exercises when pain was tolerable (10 reps × 3/day). | Standard below-knee cast applied per routine procedures. After removal, non-weight-bearing active ankle exercises were initiated. |
| Bayram 2020 | Adults >18 years with acute zone 1 fifth metatarsal fracture | 29.2 | 31.7 | Double-layer elastic bandage applied by the same investigator | Plaster immobilization |
| Lehtonen 2003 | Patients with acute displaced or unstable ankle fractures (AO-Weber type A or B) within 72h of injury, treated surgically | 26 | 27 | Functional Air-Stirrup ankle brace (Aircast) worn immediately post-op; followed same weight-bearing and exercise protocol as cast group | Below-knee cast with crutches for first 2 weeks; switched to short-leg walking cast (fiberglass) allowing partial weight-bearing for 4 weeks; full weight-bearing allowed at 6 weeks. Active/passive exercises started immediately in both groups. |
| Egol 2000 | Skeletally mature patients with isolated closed ankle fractures requiring surgical fixation | NA | NA | Removable functional brace (Aircast); active/passive ankle and subtalar exercises with physiotherapist. After discharge, brace was removed 3×/day for home exercises. | Fiberglass short-leg cast. Physical therapy began at 6 weeks post-op. Both groups began weight-bearing at 6 weeks, except syndesmotic screw patients (8 weeks). |
| Kearney 2019 | Adults with ankle fractures | 28.7 | 30.1 | Removable fixed-angle orthotic brace (typically plastic boot with padding and Velcro); exercise sheet (2 non-weight-bearing mobility exercises) provided; exercises 3×/day (10 reps each); logbook used to monitor adherence | Immobilized with plaster or fiberglass cast. |
| Stassen 2023 | Patients ≥16 years with isolated Weber B fractures (Lauge-Hansen pronation-external rotation stage 2–4A) | NA | NA | Walking boot (Rebound® Air Walker, Össur); weight-bearing as tolerated; instructed to remove boot during rest to perform ankle exercises | Below-knee cast (tibia to toes) with ankle in neutral plantargrade; non-weight-bearing strictly enforced; cast changed if discomfort occurred |
| Berg·2018 | Adults aged 18–70 years with isolated lateral malleolar fractures at syndesmosis level | 24.4 | 26.4 | Full weight-bearing as tolerated, crutches used as needed | Full weight-bearing as tolerated, crutches used as needed |
| Kortekangas 2019 | Skeletally mature patients with isolated stable Weber B fibular fractures | NA | NA | Brace worn as needed for comfort; removed when necessary; ankle movement encouraged | Below-knee cast: 3-week group had cast removed at follow-up and allowed to weight-bear as tolerated; 6-week group received new cast for continued immobilization |
| Dehghan 2016 | Acute unstable unilateral ankle fractures requiring surgical fixation | NA | NA | Orthopedic boot worn from 2 weeks post-op; immediate full weight-bearing as tolerated; boot removed 4×/day for ROM exercises; physical therapy provided; weaned off boot over 2–4 weeks starting week 6 | Below-knee cast applied at 2 weeks post-op with non-weight-bearing for 4 more weeks (total 6 weeks); cast removed at week 6, followed by active ankle exercises and full weight-bearing with boot; boot use tapered over 2–4 weeks under PT supervision |
| Jaragh 2023 | Adults >18 years undergoing surgery within 72h after unilateral ankle fracture | NA | NA | Well-padded Paris plaster posterior below-knee splint for 3 weeks; replaced by removable ankle brace to allow early physical therapy | Well-padded Paris plaster posterior below-knee splint |
| Vioreanu 2007 | Patients with acute closed ankle fractures requiring open reduction and internal fixation | 26.8 | 28.8 | After suture removal, a custom-made removable below-knee fiberglass brace was used while maintaining a non-weight-bearing status. Ankle exercises were performed three times a day for 10 minutes each session. The exercise program included active and passive range-of-motion exercises of the ankle and subtalar joints (including flexion, extension, circumduction, inversion, and eversion in both open and closed kinetic chain movements) without the brace, under the supervision of a certified physiotherapist. | Postoperatively, a non-removable below-knee fiberglass cast was applied, and the patient remained non-weight-bearing for 6 weeks. |

NA = Not available; ROM = Range of motion; PT = Physical therapy.

**eFigure1. The mean time of RTW between Removable brace and Cast immobilization.**


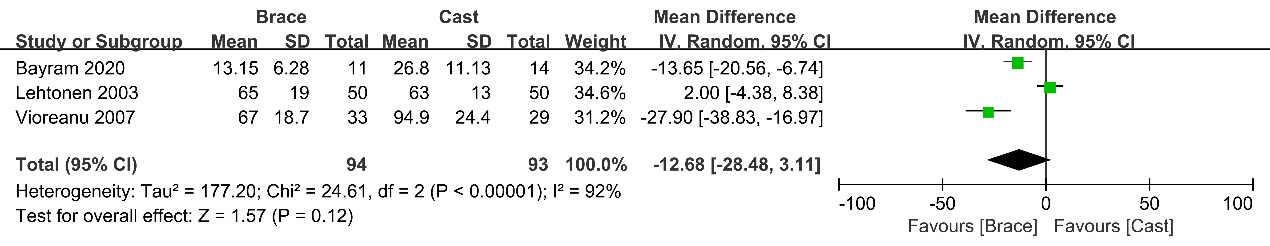


**eFigure2. Subgroup analysis: Early WB OMAS scores at 6 weeks (A),12 weeks (B); Late WB OMAS scores at 6 weeks (C),12 weeks (D).**


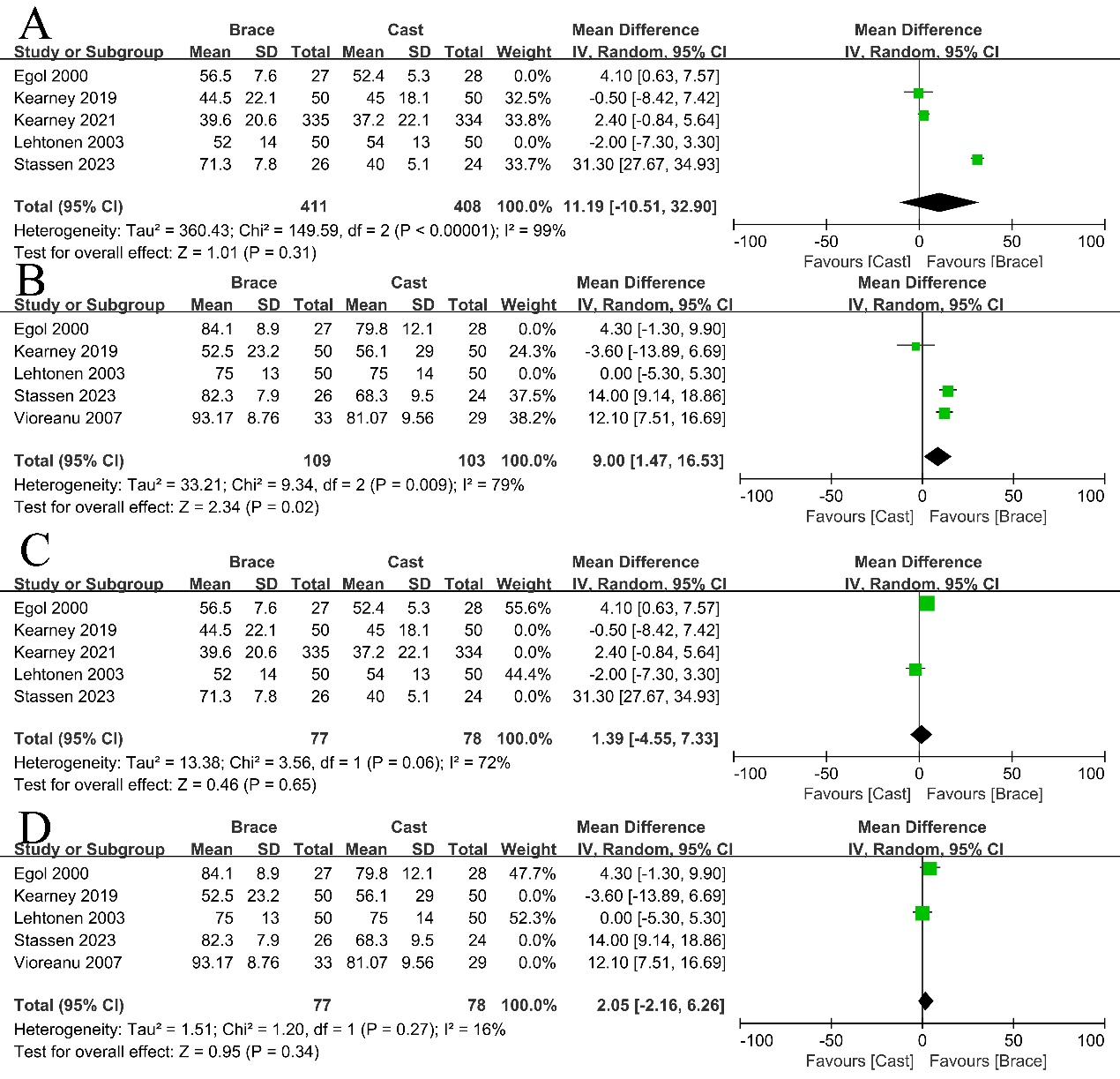


**eFigure3. Subgroup analysis: Operative patients’ OMAS scores at 6 weeks (A), 12 weeks (B), 24 weeks (C), 1 year (D).**


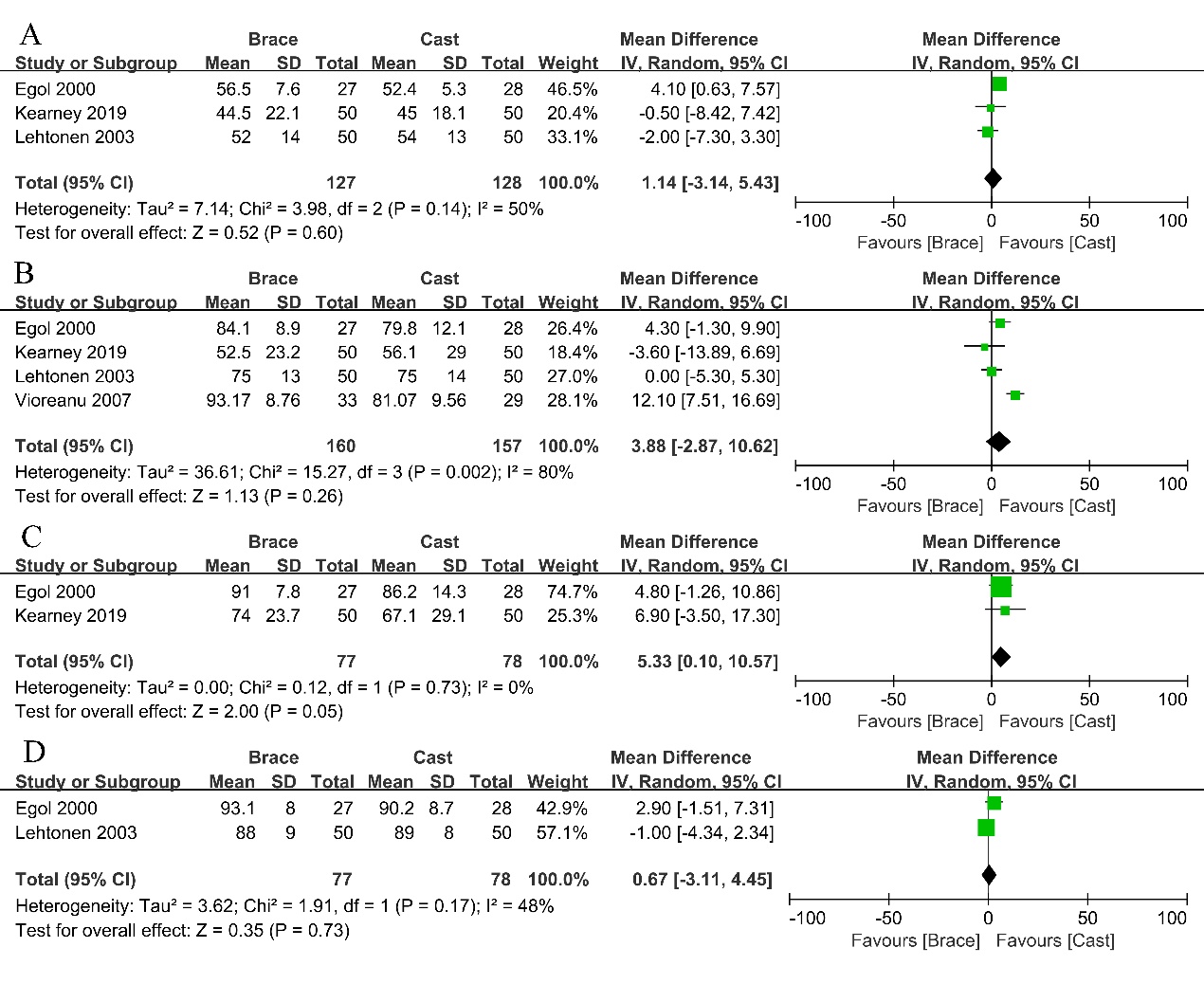


**eFigure4. Subgroup analysis: Nonoperative patients’ OMAS scores at 6 weeks (A), 12 weeks (B) and VAS scores at 6 weeks (C), 12 weeks (D).**


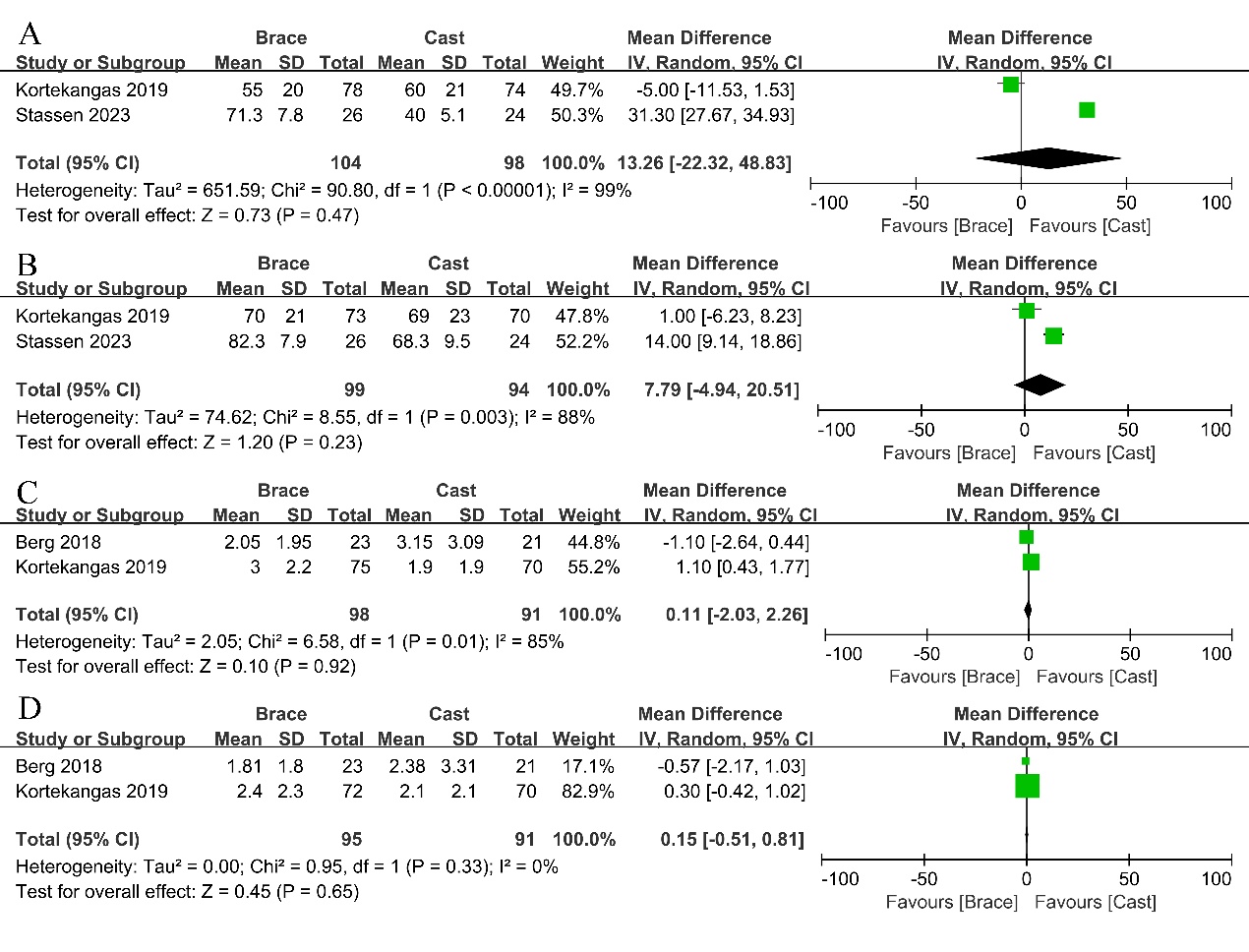


**eFigure5. Subgroup analysis: Non-adjustable group’s OMAS scores at 6 weeks (A), 12 weeks (B) and Adjustable group’s OMAS scores at 6 weeks (C), 12 weeks (D).**


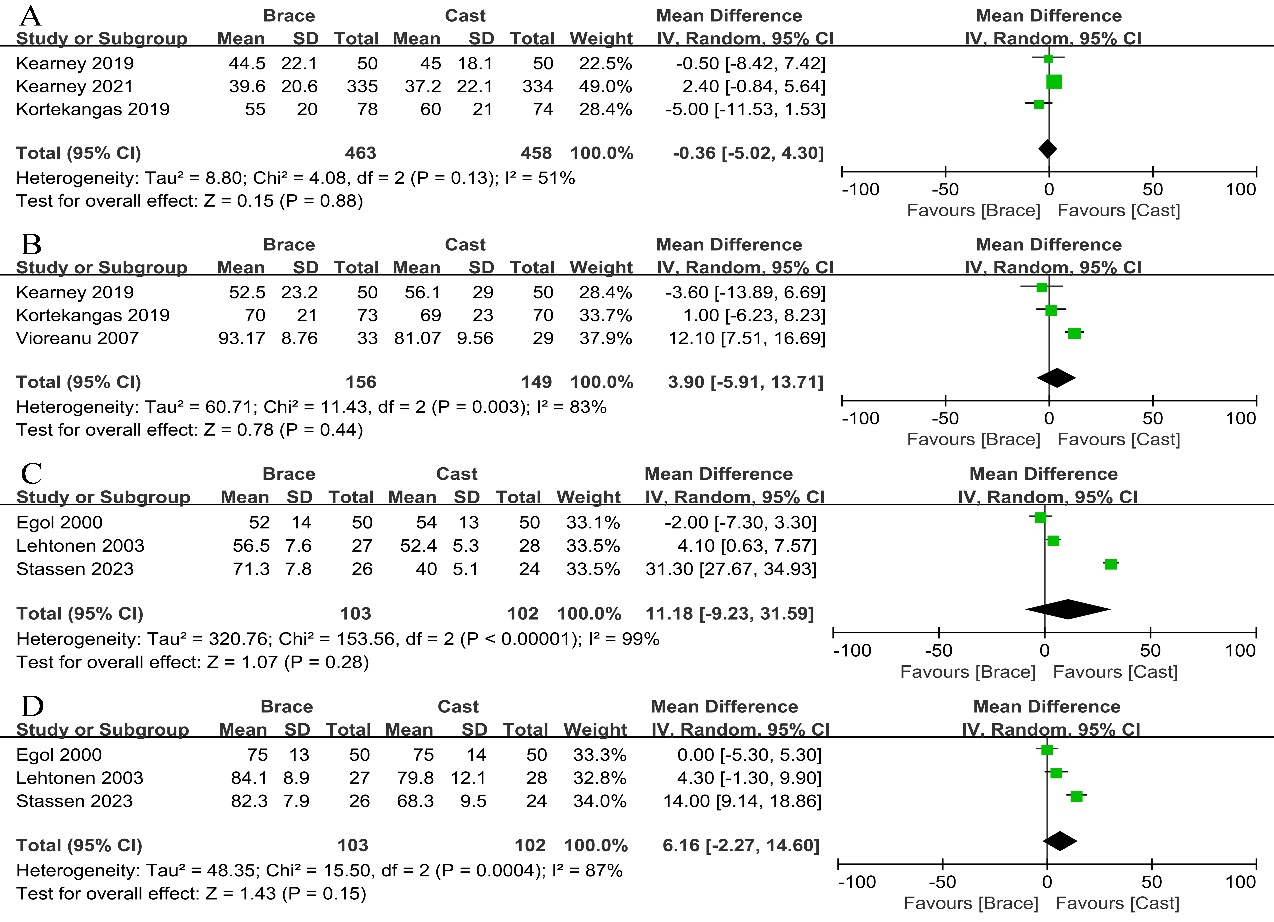

Supplement: Supplementary file 1 [file Supplementary_file_1.docx]
